# Toripalimab plus cetuximab combined with radiotherapy in a locally advanced platinum-based chemotherapy-insensitive nasopharyngeal carcinoma patient: a case report

**DOI:** 10.3389/fonc.2024.1383250

**Published:** 2024-05-10

**Authors:** Ying Piao, Yao Yang, Shihai Wu, Ling Han

**Affiliations:** ^1^ Department of Radiation Oncology, Shenzhen People’s Hospital (The Second Clinical Medical College, Jinan University, The First Affiliated Hospital, Southern University of Science and Technology), Shenzhen, China; ^2^ Department of Otorhinolaryngology, Shenzhen People’s Hospital (The Second Clinical Medical College, Jinan University, The First Affiliated Hospital, Southern University of Science and Technology), Shenzhen, China

**Keywords:** nasopharyngeal carcinoma, toripalimab, cetuximab, radiotherapy, platinum-insensitive

## Abstract

**Background:**

Locoregionally advanced nasopharyngeal carcinoma (NPC) is an epithelial malignancy that primarily occurs in East and Southeast Asia, and it is associated with relatively poor overall survival (OS). Currently, there is no reliably effective standard treatment for NPC that progresses after first-line therapy with platinum-based chemotherapy.

**Case report:**

A 55-year-old woman diagnosed with stage IVa NPC received two cycles of platinum-based chemotherapy but encountered an increase in the size of cervical lymph nodes and suffered from adverse events. The patient was then switched to toripalimab plus cetuximab combined with radical radiotherapy and had a complete clinical response within 2 months following the completion of radiotherapy without severe treatment-related adverse events.

**Conclusion:**

This case report showed that toripalimab plus cetuximab combined with radiotherapy for the treatment of patients with locoregionally advanced nasopharyngeal carcinoma may result in a fast and durable response with a manageable safety profile.

## Introduction

Nasopharyngeal carcinoma (NPC) is known as an epithelial malignancy that originates from the nasopharyngeal mucosa lining, most occurred in east and southeast Asia (1). Because of the atypical symptoms, more than 70% of NPC patients received a diagnosis of locoregionally advanced NPC upon presentation (1). However, according to the 8th Edition of the AJCC/UICC staging system for NPC in the era of Intensity-modulated radiotherapy (IMRT), the subgroup with locally advanced (LA) stage had a 5-year overall survival (OS) of 70%, which is significantly worse than patients with early stage (5-year OS of 92-98%) (2). In this regard, managing NPC with locally advanced stage poses a challenge for clinical oncologists. Currently, induction chemotherapy followed by concurrent chemoradiotherapy has been the standard treatment in LA-NPC (3, 4). Platinum-based chemotherapy is the cornerstone for LA-NPC during the induction phase (5). There is currently no reliably effective standard treatment for NPC that progresses after first-line therapy with platinum-based chemotherapy. Here we report a case of a patient with LA-NPC presented a stable disease (SD) on platinum-based chemotherapy, who achieved a complete response (CR) with toripalimab plus cetuximab in combination with radical radiotherapy.

## Case report

**Figure 1:**
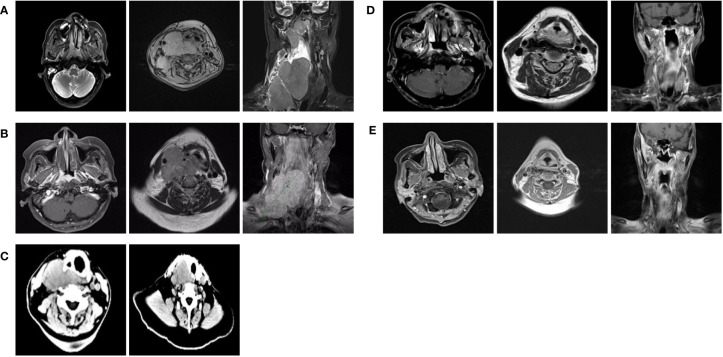
**(A)** MRI of the nasopharynx and lymph node before treatment (July, 2022). **(B)** MRI of the nasopharynx and lymph node after 2 two cycles of GPchemotherapy regimen (August, 2022). **(C)** CT of the lymph node after loading dose of toripalimab along with cetuximab (August, 2022). **(D)** MRI of the nasopharynx and lymph node after radiotherapy (October, 2022). **(E)** MRI of the nasopharynx and lymph node performed 2 months after the completion of radiotherapy (December, 2022).

In July 2022, a 55-year-old woman complained of a 5-month history of a progressively enlarging cervical mass with pain. Physical examination was unremarkable except for the presence of large palpable masses in the neck associated with tenderness. Nasal endoscopy indicated the presence of fleshy masses on the right lateral and posterior walls of the nasopharynx. Biopsies obtained from the nasopharynx confirmed a diagnosis of nonkeratinizing undifferentiated NPC. Magnetic resonance imaging (MRI) of the nasopharynx and neck examination revealed the presence of nasopharyngeal lesions and enlarged bilateral cervical lymph nodes (level II,III,IV,V on the right and level II on the left) fused into masses, with the maximum dimension measuring 11.4cm in the coronal plane (**Figure 1A**). Chest and abdominal enhanced computed tomography (CT) as well as whole-body bone scan showed no signs of tumor metastasis. The patient was then diagnosed with nasopharyngeal nonkeratinizing undifferentiated cell carcinoma, classified as T3N3M0 (stage IVa) according to the 8th edition of the AJCC staging system. The patient received two cycles of the GP chemotherapy regimen (gemcitabine 1000mg/m2, D1,8; cisplatin 75mg/m2, D1; 21 days per cycle). The symptom of swallowing difficulty emerged after 2 cycles of chemotherapy. Subsequent MRI scan indicated a size reduction in the nasopharyngeal lesions but an enlargement in the cervical lymph nodes (**Figure 1B**). What’s more, two cycles of chemotherapy resulted in significant adverse events, including grade 3 anemia, grade 1 leukopenia, grade 1 thrombocytopenia, and grade 2 renal dysfunction (according to the NCI CTCAE v5.0) (6).

To further elucidate the nature of the enlarged cervical lymph nodes, an additional fine needle aspiration was performed. The histopathological analysis confirmed lymph node metastases of nasopharyngeal carcinoma, and immunohistochemical assessment revealed epidermal growth factor receptor (EGFR) expression at 90% intensity with a score of 3+ and programmed death-ligand 1 (PD-L1) combined positive score (CPS) of 50.

**Figure 2:**
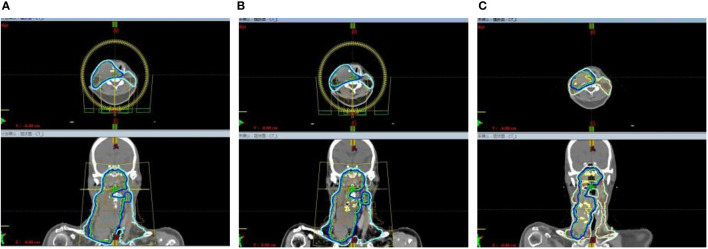
Due to the rapid reduction in the size of cervical lymphnodes, adjustment to the radiotherapy plan were made every two weeks. **(A)** The beam arrangement and isodose distribution at the beginning of radiotherapy. **(B)** The beam arrangement and isodose distribution in the third week of radiotherapy. **(C)** The beam arrangement and isodose distribution in the fifth week of radiotherapy.

**Figure 3:**
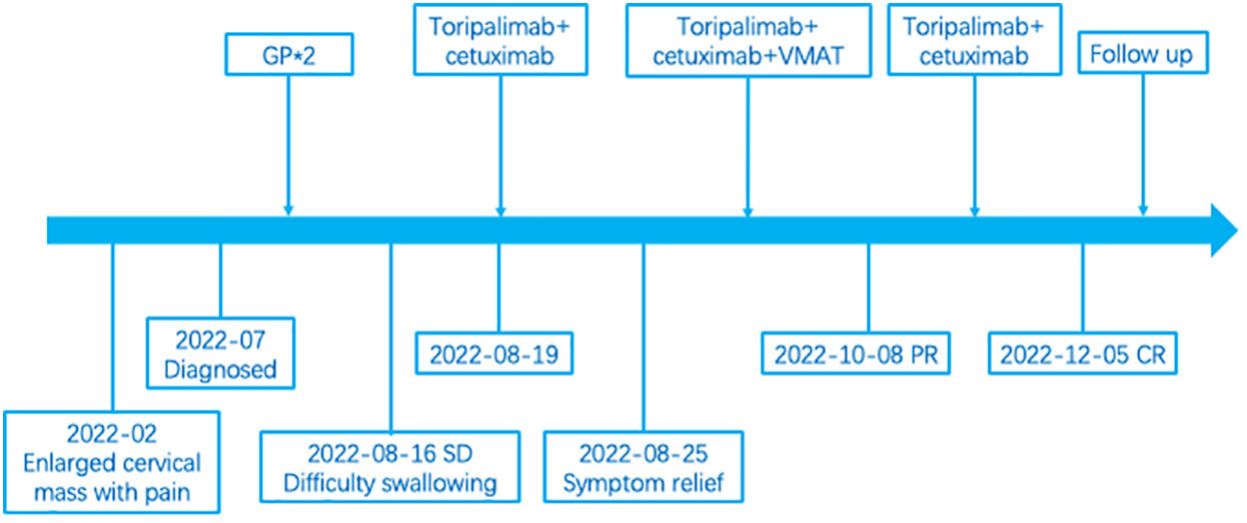
The time line of interventions and patient outcomes.

Considering the intolerability of chemotherapy, the patient underwent toripalimab plus cetuximab combined with volumetric modulated arc therapy (VMAT) from August 2022. One week after the loading dose of toripalimab (240mg every 3 weeks) along with cetuximab (400mg/m2, then weekly administration of 250 mg/m2), the patient reported a significant improvement in neck swelling and swallowing difficulty compared to the previous condition. Subsequent CT scans showed a slight reduction in tumor size (**Figure 1C**). Then the radiotherapy was started in August-25 2022, with the following dose distribution: 69.96Gy/33F to the gross tumor, 66Gy/33F to the cervical lymph nodes, 60Gy/33F to the planning target volume 1, and 50.1Gy/33F to the planning target volume 2, 5 times per week. Target contouring was performed referring to the Chinese Society of Clinical Oncology (CSCO) guideline and international guidelines for nasopharyngeal carcinoma (7, 8). Intravenous toripalimab and cetuximab were given concurrently. Notably, due to the rapid reduction in cervical lymph node size, adjustments to the radiotherapy plan were made every two weeks (**Figure 2**). Post-treatment MRI scans suggested partial remission (PR) (**Figure 1D**). After radiotherapy, the patient continued toripalimab (240mg every 3 weeks) along with cetuximab (250 mg/m2 every week) until 2 months later when the MRI of nasopharynx and neck examination indicated CR (**Figure 1E**). The patient experienced grade 1 nausea and grade 2 oral mucositis during RT. Then she was regularly followed up with periodical imagological examinations and remained disease free until the time of writing this report, without further therapies. The timeline depicting interventions and outcomes of the case is illustrated in **Figure 3**.

## Discussion

Despite advancements, LA-NPC remains a significant challenge for clinical oncologists and patients. A study published in 2019 (9) showed that induction chemotherapy with the regimen of GP added to chemoradiotherapy demonstrated a significant enhancement in both recurrence-free survival and OS in patients with LA-NPC. Another study (10) demonstrated that GP and TPF (docetaxel, cisplatin, and fluoropyrimidines) regimens showed equivalent efficacy to the TP (docetaxel and cisplatin) regimen in addressing primary nasopharyngeal tumors after induction chemotherapy, and GP exhibited the highest overall response rate (ORR) when it came to cervical lymph nodes. However, in this case, the patient exhibited cervical lymph node enlargement after two cycles of GP chemotherapy.

The antitumor activity and safety of immune checkpoint inhibitors (ICIs) targeting programmed death 1 (PD-1), including pembrolizumab (11), nivolumab (12), toripalimab (13), and others (14), have been well-established in studies involving patients with recurrent or metastatic (R/M) NPC, showing an ORR of 20-30%. Among these ICIs, the promising efficacy of toripalimab in combination with IMRT for unresectable recurrent NPC was demonstrated, with a disease control rate (DCR) of 95.8% achieved at 3 months post-radiotherapy, and a 1-year progression-free survival (PFS) of 91.8% (15). Theoretically, radiotherapy has the potential to not only alter the tumor microenvironment (TME) by modifying the peptide composition and enhancing the expression of major histocompatibility complex (MHC) class 1 but also to activate the DNA double-strand break repair pathway and elevate PD-L1 expression in cancer cells (16, 17). This provides a compelling rationale for the combination of radiotherapy and PD-1 therapy.

Cetuximab also proves to be an effective agent when used in combination with both IMRT and radiochemotherapy for NPC patients (18, 19). Cetuximab exerts its antitumor impact by inhibiting epidermal growth factor receptor (EGFR) signaling and activating the natural killer (NK) cell-mediated antibody-dependent cell cytotoxicity (ADCC). Furthermore, cytokines released by activated NK cells increase CD-8+ T-cell-mediated destruction of tumor cells and promote the recruitment of other immune cells to the TME. However, this immune stimulatory activity can also lead to immunosuppressive feedback loops, including upregulated expression of PD-L1 on cancer cells and the recruitment of immunosuppressive regulatory T cells and myeloid-derived suppressor cells (MDSC) (20). Given the distinctive mechanisms of cetuximab and ICIs, there is a robust theoretical foundation for combining cetuximab with ICIs for the treatment of cancer, as ICIs can overcome these immunosuppressive counter-mechanisms in the TME.

In an earlier phase II study focused on recurrent or metastatic head and neck squamous cell carcinoma (R/M HNSCC), the combination of cetuximab and pembrolizumab demonstrated promising results, with a favorable ORR of 45% and a median overall survival (mOS) of 18.4months (21), exceeding published response rates with pembrolizumab (16–18%) (22–24) or cetuximab (6–13%) (25, 26) monotherapy. Another study investigating the combination of cetuximab and nivolumab also demonstrated a promising ORR ranging from 22% to 37% (27). Toripalimab, a humanized IgG4 monoclonal antibody targeting PD-1, has demonstrated its effectiveness and safety in the treatment of R/M NPC either as a standalone therapy or in combination with chemotherapy or IMRT (13, 15, 28). Accordingly, the combination of toripalimab with cetuximab could synergize, and is likely to achieve a clinical benefit in LA-NPC. In this case, the patient exhibited a notable improvement in symptoms after receiving the loading dose of toripalimab with cetuximab, despite being insensitive to platinum-based chemotherapy. Even more surprisingly, during radiotherapy, the tumor displayed a rapidly reduction in size, achieving complete remission (CR) after 2 months of radiotherapy, with no severe treatment-related adverse events. Up to now, the tumor response of CR has been maintained during the 12-month follow-up after treatment.

Nowadays, there are few studies focusing on LA-NPC that is insensitive to induction chemotherapy, especially for patients who develop SD or progressive disease (PD) after undergoing this treatment. A study (29) published in 2024 recruited 56 NPC patients at stage III-IVa who exhibited SD or PD following platinum-based induction chemotherapy. After treatment with nimotuzumab, an anti-EGFR monoclonal antibody, in combination with chemoradiotherapy, the ORR for involvement of the nasopharynx and cervical lymph nodes were 98.2% and 98.1%, respectively. To our knowledge, there have been no reports on the efficacy and safety of combining ICIs with cetuximab for use in conjunction with radical radiotherapy in NPC patients.

In summary, after exhibiting insensitivity to platinum-based chemotherapy, this patient with LA-NPC benefited from this comprehensive treatment, achieving significant clinical improvement even in a generally less favorable clinical condition. Of course, It’s important to note that findings from a single case report cannot be applicable for all patients with LA-NPC because of potential for publication bias. Whether the response could be attributed to toripalimab alone or to the combination of toripalimab and cetuximab still requires further confirmation. The results of randomized controlled clinical trials are crucial for guiding treatment decisions. Continued research to identify the optimal modalities and sequence for individual patients with LA-NPC is still needed.

## Data availability statement

The original contributions presented in the study are included in the article/supplementary material. Further inquiries can be directed to the corresponding author.

## Ethics statement

The studies involving humans were approved by The Ethics Committee of Shenzhen People’s Hospital. The studies were conducted in accordance with the local legislation and institutional requirements. The participants provided their written informed consent to participate in this study. Written informed consent was obtained from the individual(s) for the publication of any potentially identifiable images or data included in this article.

## Author contributions

YP: Data curation, Methodology, Formal analysis, Resources, Writing – original draft. YY: Data curation, Formal analysis, Writing – original draft. SW: Conceptualization, Investigation, Project administration, Software, Supervision, Writing – review & editing. LH: Data curation, Methodology, Software, Validation, Writing – review & editing.
